# Do stress and anxiety in early pregnancy affect the progress of labor: Evidence from the Wirral Child Health and Development Study

**DOI:** 10.1111/aogs.14063

**Published:** 2021-02-05

**Authors:** Pauline Slade, Kayleigh Sheen, Andrew Weeks, Susan Wray, Leonardo De Pascalis, Karen Lunt, Carol Bedwell, Belinda Thompson, Johnathan Hill, Helen Sharp

**Affiliations:** ^1^ Department of Primary Care and Mental Health Institute of Population Health Sciences University of Liverpool Liverpool UK; ^2^ School of Psychology Faculty of Health Liverpool John Moores University Liverpool UK; ^3^ Department of Women’s and Children’s Health University of Liverpool Liverpool Women’s Hospital Liverpool UK; ^4^ Department of Women’s and Children’s Health University of Liverpool Liverpool UK; ^5^ Psychological Sciences Institute of Health and Life Sciences University of Liverpool Liverpool UK; ^6^ Department of Midwifery, Child and Reproductive Health University of Chester Chester UK; ^7^ International Public Health Liverpool School of Tropical Medicine Liverpool UK; ^8^ Arrowe Park Hospital Birkenhead UK; ^9^ School of Psychology and Clinical Language Sciences University of Reading Reading UK

**Keywords:** anxiety, augmentation, epidural, fear of childbirth, labor duration, pregnancy‐specific stress

## Abstract

**Introduction:**

Despite widespread belief that anxiety causes longer labor, evidence of association is inconsistent. Data gathered as part of a prospective epidemiological longitudinal study were used to investigate associations between antenatal anxiety and pregnancy‐specific stress, and labor progression was assessed by duration and use of augmentation.

**Material and methods:**

Pregnant primiparous women completed measures for anxiety and pregnancy‐specific stress at 20 weeks’ gestation (n = 1145). Birth outcome data were extracted from medical records. Regression analyses and a path analysis assessed associations between antenatal anxiety and pregnancy‐specific stress, and indices of labor progression (labor duration and augmentation).

**Results:**

Anxiety/pregnancy‐specific stress were not directly associated with duration of stage 1 labor (HIGH/LOW anxiety: mean difference = 13.94 minutes, SD = 20.66, 95% CI −26.60 to 54.49, *P* < .50)/(HIGH/LOW pregnancy‐specific stress: mean difference = 12.05 minutes, SD = 16.09, 95% CI −19.52 to 43.63, *P* < .45). However, anxiety/pregnancy‐specific stress were associated with epidural use (HIGH/LOW anxiety: 39% vs 31%, *P* < .042; HIGH/LOW pregnancy‐specific stress: 38% vs 29%, *P* < .001), which was itself associated with longer labor (mean difference: 158.79 minutes, SD = 16.76, 95% CI 125.89‐191.68, *P* < .001). Anxiety and pregnancy‐specific stress were associated with increased likelihood of augmentation but these associations were nonsignificant after accounting for epidural, which was itself highly associated with augmentation. However, path analysis indicated an indirect effect linking pregnancy‐specific stress, but not general anxiety, to labor duration and augmentation: elevated pregnancy‐specific stress led to greater use of epidural, which was linked to both increased rates of augmentation, and increased labor duration.

**Conclusions:**

Contrary to general belief, general anxiety and specific pregnancy stress were not directly linked to longer duration of stage one labor. However specific pregnancy stress was associated with epidural use, which in turn was significantly associated with risk of augmentation, and longer stage one labor. Identification of pregnancy‐specific stress could help to identify women for whom psychological interventions could improve birth experience.

**Abbreviations**
BMI: body mass index
CI: confidence interval
FOC: fear of childbirth
IMD: Index of Multiple Deprivation
OR: odds ratio
PSS: Pregnancy Stress Scale
STAI: State‐Trait anxiety inventory–state version19
WCHADS: Wirral Child Health and Development Study

**Key message**

Higher anxiety/pregnancy‐specific stress was not directly associated with longer labor. Both predicted epidural use, which was associated with augmentation and longer labor. Only pregnancy‐specific stress demonstrated a clear pathway via epidural to both augmentation and longer labor duration.

## 1 INTRODUCTION

Despite widespread belief that maternal stress causes longer labor, evidence of association between antenatal anxiety and rate of progress in labor is inconsistent.^1^, ^2^ Prolonged labor may require obstetric intervention, which can negatively influence women’s childbirth experience.^3^, ^4^, ^5^

Anxiety in pregnancy can include general anxiety, pregnancy‐specific stress, or specific fear of childbirth (FOC). Adams et al^6^ found that, at 32 weeks' gestation, FOC, but not general anxiety, was independently associated with 47 minutes’ longer labor. Reck et al^7^ found similar patterns but with pregnancy‐specific stress, not general anxiety, at 24 weeks being related to total labor time. Conversely, Sluijs et al^8^ suggest that neither anxiety nor FOC measured at 30 weeks had any association with the birth‐giving process, including the first stage of labor, although the power of the study might be compromised. Large birth cohort study samples also found mixed results. Laursen et al^9^ suggested that FOC (assessed by a single question repeated in both first and third trimesters) was associated with “protracted labor” in nulliparous women. Koelewijn et al,^8^ utilizing very large samples and solely first trimester measures, found FOC did show some association with stage one, whereas general anxiety did not. However, the labor duration measure was acknowledged as insensitive, being categorized by 6‐hour blocks. Their research did suggest anxiety was associated with pain relief and sedation. Hall et al^10^ found that FOC but not general anxiety (when measured at 35‐39 weeks of gestation) predicted use of epidural. Some of these complexities of findings may relate to timing and focus of the measures, parity and the way duration of labor is assessed. Overall, pregnancy‐specific anxiety or FOC rather than general anxiety appears more likely to be associated with labor duration.

Slow progress in the active phase of labor is generally augmented with oxytocin.^11^ FOC late in pregnancy has been associated with increased likelihood of augmentation.^12^ Slow labor can also result in emergency cesarean section but, again, an association between general/pregnancy‐specific anxiety and emergency cesarean section is not consistently reported.^10^, ^13^, ^14^ If early antenatal anxieties do predict labor progression, then identification allowing timely psychological intervention in pregnancy could reduce the risk of prolonged labor and/or associated interventions, improving birth experiences and reducing postnatal psychological difficulties.^15^, ^16^ The role of epidural, given its associations with antenatal anxiety and stress^1^ and certain indicators of labor progression,^6^ also requires examination.

The aims and objectives were to investigate whether, controlling for epidural use, general anxiety or pregnancy‐specific stress at 20 weeks of gestation:

- predicts duration of first stage of labor (*hypothesis 1*),
- predicts use of augmentation (*hypothesis 2*),

## 2 MATERIAL AND METHODS

Data were gathered as part of a UK Medical Research Council‐funded prospective epidemiological longitudinal study of emotional, psychological, social and biological predictors of child development, the Wirral Child Health and Development Study (WCHADS). Data from the study are listed by the UK Medical Research Council Cohort Directory to maximize public benefit from datasets gathered through public funding. Hypotheses were formulated by the research team, including the two WCHADS principal investigators, prior to any analysis of data relating to these questions.

### 2.1 Participants and procedure

Women were having their first baby, aged 18 or above, booked for antenatal care at 12 weeks of gestation between 12 February 2007 and 29 October 2008 at the Wirral University Teaching Hospital.^17^ This was a consecutive sample of first‐time pregnant mothers registering for antenatal care. Wirral socioeconomic conditions range between the deprived inner city and affluent suburbs, but with low numbers of women from ethnic minorities. Clinic midwives approached women attending their 20 weeks of gestation screening to ask for their agreement to speak with one of three research midwives. After obtaining written informed consent, the study midwives administered questionnaires and subsequently gathered obstetric outcome data from medical records.

**FIGURE 1:**
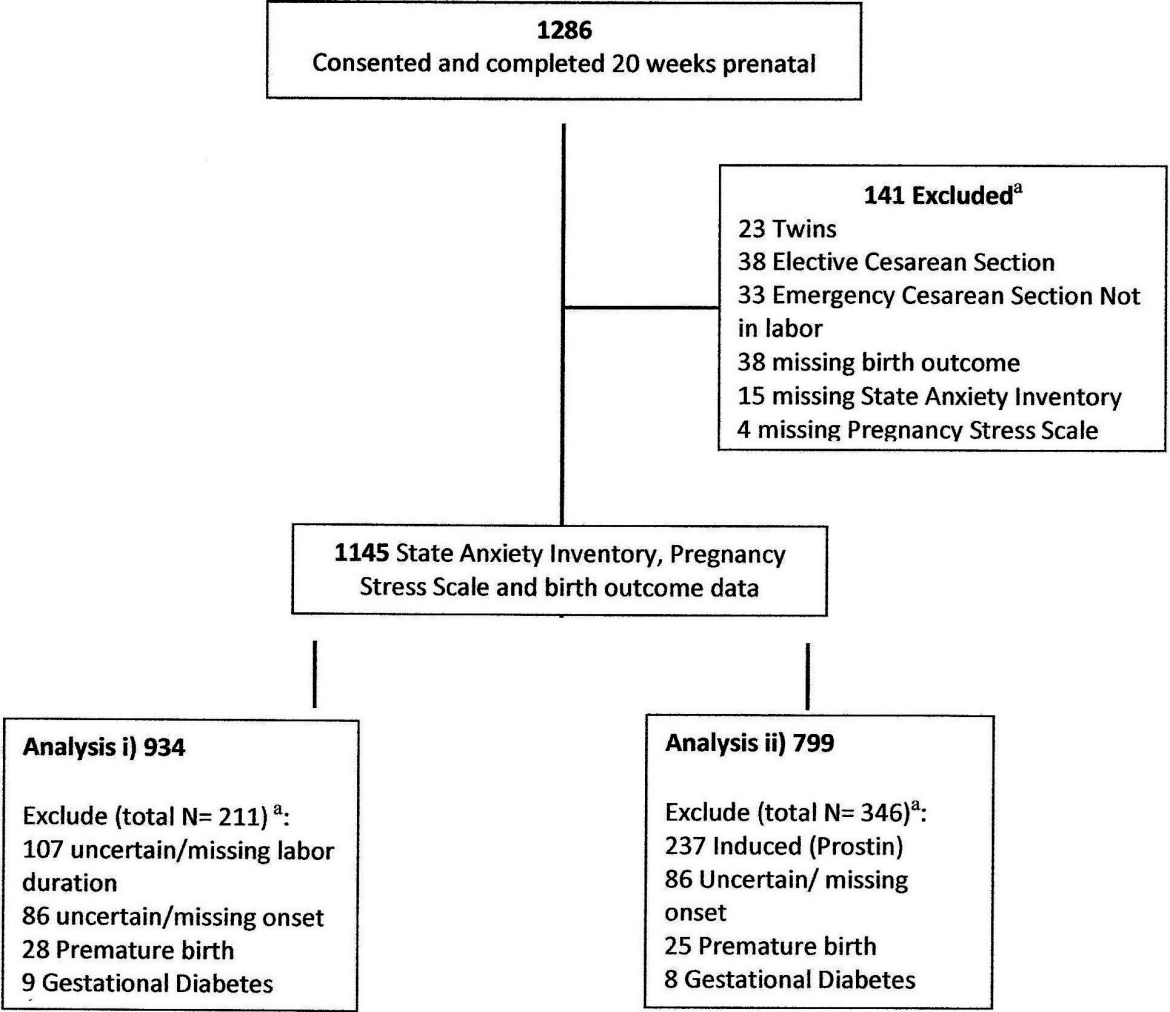
Sampling and inclusion for each analysis. ^a^Categories are not mutually exclusive

Of the sample, 1286 provided antenatal data at 20 weeks of gestation. This represents a response rate of 68% from a potential sample of 1891 women. For the analysis, women with twin births, emergency cesarean section occurring when not in labor, and elective cesarean sections were excluded, the latter two groups because these women did not experience labor. After excluding cases with missing data on required variables, the final sample was 1145 women (Figure 1).

### 2.2 Measures

#### 2.2.1 Demographics

Demographics included age (years) and body mass index (BMI). Socioeconomic status was determined using the revised English Index of Multiple Deprivation (IMD),^18^ which assigns a score from least (IMD 32, 482) to most deprived (IMD 1). Mothers in the current sample were assigned an IMD rank based on their postcode and a quintile based on the UK distribution of deprivation.

#### 2.2.2 State‐Trait Anxiety Inventory – state version (STAI)

The state version consists of 20 items on a 4‐point scale assessing anxiety, ie fear, nervousness, discomfort in the present, as this was the variable of interest rather than trait anxiety or general tendencies.^19^ Scores range between 20 and 80, with higher scores indicating greater anxiety. Cronbach’s Alpha for the current study was excellent at 0.92.

Scores were dichotomized at 40 to indicate clinical anxiety (≥40) and nonclinical anxiety (≤39), in line with previous studies with pregnant women (sensitivity 80.95%, specificity 79.75%, positive predictive value 51.5% and negative predictive value 94%).^20^

#### 2.2.3 Pregnancy Stress Scale (PSS)

This included four items assessing feelings about pregnancy using a 5‐point scale.^21^, ^22^ Cronbach’s Alpha for this scale was acceptable at 0.82. Scores were dichotomized using the median (5.00) to infer high (>5) and low (≤5) pregnancy‐related stress.

#### 2.2.4 Obstetric record

The following variables were extracted from the obstetric records and, if necessary, were verified by a consultant obstetrician (AW):

*Mode of birth:* Vaginal, ventouse, forceps, emergency cesarean section (all based on midwife case notes).

*Epidural*: Coded Yes/No.

*Induction*: Receipt of prostaglandin induction agents (mechanical induction methods were not in use at that time) with/without oxytocin or artificial rupture of membranes. To create two homogeneous, clearly separated datasets, women who only required artificial rupture of membranes and/or oxytocin to initiate labor, with no prostaglandins, were excluded from analysis as, according to the operationalized definition, they were neither fully induced nor did they spontaneously begin labor.

Women who received no prostaglandins, artificial rupture of membranes or oxytocin were defined as spontaneously starting labor.

*Augmentation*: All women who spontaneously began labor (absence of prostaglandins/artificial rupture of membranes/oxytocin) but who subsequently received oxytocin for augmentation.

*Duration of the first stage of labor*: Time from the onset of regular painful contractions to the full dilation of the cervix, recorded by the midwife providing care.

### 2.3 Statistical analyses

Data were analyzed using SPSS 19 (IBM Corp.). Bivariate associations were assessed using Pearson’s correlation (*r*), *t* test and chi‐square analyses. Analyses involving the STAI and PSS are presented using dichotomized scores; analyses on continuous STAI and PSS scores showed identical patterns of findings and so are not presented.

For hypothesis 1: A stepwise multivariate regression analysis entering potentially confounding variables (BMI, epidural, induction) at block one, STAI at block two and PSS at block three.

For hypothesis 2: Two sequential logistic regression analyses were planned to assess predictive utility of either STAI score or PSS alongside epidural use. For both analyses, either STAI or PSS was entered at block 1 and epidural at block 2. Contributions of individual coefficients in each model block were assessed using the Wald test statistic.^23^ Odds ratios (OR) with 95% confidence intervals (95% CI) are presented.

Indirect effects related to the models in the first two hypotheses were investigated using a path analysis model, conducted in MPLUS 7 (www.StatModel.com). Bias‐corrected bootstrap (5000 samples) was used to estimate indirect effects.^24^

### 2.4 Ethical approval

Ethical approval was granted by the Cheshire North and West Research Ethics Committee on 27 June 2006 (REF 05/Q1506/107).

## 3 RESULTS

### 3.1 Demographic and birth outcome data

Table 1 shows demographic and birth outcome data. Approximately 42% of women (n = 474) were in the most deprived UK IMD quintile. Mothers were slightly below average age for first‐time mothers (mean = 27.5 years) in England and Wales.^25^ National birth statistics for England 2008‐2009 indicate that 17% of women received an epidural, 63% had spontaneous vaginal deliveries, 6% needed use of forceps, 7% ventouse, and there was a 15% emergency cesarean section.^26^ Comparatively, women in this study experienced higher rates of epidural, ventouse and use of forceps, although national statistics are not directly comparable, as they also include multiparous women.

**TABLE 1:** Participant characteristics and birth outcome data

	n	Mean (SD)
Age	1145	26.7 (5.7)
BMI	1141	26.1 (5.3)
Duration first stage,^b^ min	1038	401.55 (243.48)

	N^a^	%
Epidural	375	32.8
Preterm labor (<37 weeks of gestation)	25	2.2
Spontaneous vaginal delivery	680	59.5
Ventouse	152	13.3
Forceps	133	11.6
Emergency cesarean section	180	15.7
Induction^c^	237	22.4
Augmentation	297	25.9

^a^ Total number = 1145 unless otherwise indicated.

^b^ Duration total number = 1038 after exclusion for uncertain labor duration (n = 107).

^c^ Induction total number = 1059 after exclusion for uncertain onset (n = 86).

### 3.2 Antenatal anxiety, pregnancy‐specific stress and obstetric interventions

STAI scores (mean = 31.54, SD = 9.82) and PSS (mean = 5.66, SD = 3.36) were moderately correlated (*r* = .48, *P* < .001). In all, 211 women (18.4%) showed STAI scores exceeding the clinical cutoff. No significant difference was found, according to birth mode (vaginal, instrumental, emergency cesarean section), in the proportions of women scoring above or below threshold on the STAI (χ^2^[*df* = 2] = 3.58, *P* = .167) or the PSS (χ^2^[*df* = 2] = 4.29, *P *= .117). However, a significantly larger proportion of women with high rather than low STAI scores received an epidural (n = 82, 39% vs n = 293, 31%; χ^2^[*df* = 1] = 4.39, *P *= .042). A similar pattern emerged for PSS scores and epidural (High: n = 194, 38% vs low: n = 181, 29%; χ*^2^*[*df* = 1] = 9.75, *P *< .001).

### 3.3 Hypothesis testing

#### 3.3.1 Does antenatal anxiety or pregnancy‐specific stress at 20 weeks of gestation predict duration of the first stage of labor?

After analysis‐specific exclusions (Figure 1), data were available for 934 women. Bivariate associations between antenatal and confounding variables and labor duration were assessed (Table 2). There was no association between either STAI or PSS and labor duration. In contrast, epidural and emergency cesarean section were all associated with longer labor, whereas induction was associated with a shorter labor. As neither the anxiety variable nor BMI was associated with labor duration, they were not entered into the multivariate analysis.

**TABLE 2:** Initial *t* tests and bivariate correlations for stage 1 labor duration in minutes, anxiety and obstetric interventions

		n	Mean	SD	Test	*P*	Cohen’s *d*	Mean (SE) difference (in labor duration)	CI of difference
Emergency cesarean section	Yes	66	563.55	255.66	*t* = 5.26	<.001	0.65	161.58 (30.73)	101.26‐221.89
	No	868	401.97	239.52					
Epidural	Yes	274	525.59	275.92	*t* = 9.40	<.001	0.64	158.79 (16.76)	125.89‐191.68
	No	660	366.81	213.09					
Induction	Yes	193	356.12	296.10	*t* = −3.68	<.001	0.27	−72.18 (19.59)	−110.63 to −33.72
	No	741	428.30	226.49					
STAI	≥40	171	424.78	230.46	*t* = 0.68	.500	0.06	13.94 (20.66)	−26.60 to 54.49
	≤39	763	410.83	247.12					
PSS	>5	412	420.12	233.05	*t* = 0.75	.454	0.05	12.05 (16.09)	−19.52 to 43.63
	≤5	522	408.07	252.56					
BMI	—	934	—	—	*r* = .01	.849	—		—

Total number = 934 after application of exclusion criteria (Figure 1).

BMI, body mass index; CI, confidence interval; PSS, Pregnancy Stress Scale; STAI, State‐Trait anxiety inventory – state version 19.

In a stepwise regression (Table 3) all retained independent variables (IVs) (emergency cesarean section, epidural, induction) significantly predicted labor duration (*F*_3,930_ = 48.61, *P* < .001), accounting for 13% of the variance (adjusted *R* ^2^ = .13). Emergency cesarean section and epidural were uniquely associated with longer labor, whereas induction uniquely predicted shorter labor (see Table 3).

**TABLE 3:** Multiple linear regression analysis predicting the duration of the first stage of labor

	*B*	95% CI	SE (*b*)	β
Constant	379.10**	360.61‐397.58	9.42	
Emergency cesarean section	131.56**	73.69‐189.43	29.49	0.14
Epidural	162.99**	130.12‐195.86	16.75	0.30
Induction	−110.43**	−147.08 to −73.79	18.67	‐0.18

n = 934; *b*, unstandardized coefficient; 95% CI, 95% confidence intervals for B; SE(*b*), standard error for *b*; *β*, standardized coefficient.

** *P* < .001.

#### 3.3.2 Does antenatal anxiety or PSS at 20 weeks of gestation predict requirement for augmentation?

Figure 1 shows specific inclusion criteria for this hypothesis. Listwise deletion resulted in a sample size of 799. Bivariate associations between IVs (epidural use, BMI, STAI and PSS scores) and use of augmentation were assessed (Table 4). A significantly larger proportion of women with elevated rather than low STAI scores required augmentation (χ*^2^*[*df* = 1] = 5.44, *P* = .025, OR = 1.52). The same pattern emerged for PSS scores (χ*^2^*[*df* = 1]) = 4.27, *P* = .023, OR = 1.36), and for those receiving an epidural (n = 55; χ*^2^*[*df* = 1] = 180.26, *P* < .001). BMI and augmentation were not significantly associated, so this was not retained as a confounding variable.

**TABLE 4:** Bivariate associations between epidural, body mass index (BMI), State‐Trait anxiety Inventory (STAI) and Pregnancy Stress Scale (PSS) scores with augmentation

		STAI		PSS		Epidural		BMI
		High	Low	High	Low	Yes	No	
Augmentation	Yes	68	222	167	123	158	132	288
	No	85	424	245	264	55	454	509
**Test**		**χ^2^**		**χ^2^**		**χ^2^**		***t* test**
		5.44		6.61		180.26		1.51
*P*		.025		.012		<.001		.131

n = 799 after exclusions for induction, uncertain/missing onset of labor, premature birth and gestational diabetes (total exclusions 346).

### 3.4 STAI, epidural and augmentation

#### 3.4.1 Block 1 (STAI only)

STAI scores significantly distinguished presence and absence of augmentation (χ^2^[*df* = 1] = 5.33, *P* = .021) (Table 5). STAI scores significantly contributed to prediction (Wald[1] = 5.39, *P* = .020). The odds of requiring augmentation were 1.5 times higher for women with high STAI scores (OR = 1.53, 95% CI 1.07‐2.19). However, including STAI scores did not change the correctly identified percentage from the constant‐only model (63.7%), indicating only limited contribution of STAI to the model.

**TABLE 5:** Stepwise logistic regression predicting augmentation by (1) State‐Trait anxiety Inventory (STAI) and epidural and (2) Pregnancy Stress Scale (PSS) and epidural

			*B*	SE *B*	OR	95% CI
Model 1 (STAI)	Block 1	STAI	0.42*	0.18	1.53	1.07‐2.19
		Constant	−0.65	0.08	0.52	
	Block 2	STAI	0.18	0.21	1.20	0.80‐1.82
		Epidural	2.27**	0.19	9.72	6.75‐14.00
		Constant	−1.27	0.11	0.28	
Model 2 (PSS)	Block 1	PSS	0.31*	0.15	1.36	1.02‐1.81
		Constant	−0.70	0.10	0.50	
	Block 2	PSS	0.14	0.17	1.15	0.82‐1.60
		Epidural	2.28**	0.19	9.75	6.78‐14.03
		Constant	−1.29	0.12	0.27	

n = 799.

* *P* < .05

** *P* < .001.

#### 3.4.2 Block 2 (STAI and epidural)

The model remained significant (χ^2^[*df* = 2] = 179.04, *P* < .001). Including epidural use, correct classification increased to 76.6%. Epidural was a unique significant predictor (Wald[1] =149.67, *P* < .001) but STAI was rendered nonsignificant (Wald[1] = 0.76, *P* = .383) (Table 5). The odds of augmentation were nearly 10‐fold for women receiving an epidural (OR = 9.61, 95% CI 6.66‐13.85). Epidural use appeared to account fully for the association between anxiety and augmentation.

### 3.5 Pregnancy‐specific stress, epidural and augmentation

#### 3.5.1 Block 1 (PSS only)

The model including only PSS to predict augmentation was significant (χ^2^[*df* = 1] = 4.26, *P* = .039), with PSS significantly associated with augmentation (Wald(1) = 4.257, *P *= .039). The odds of requiring augmentation were 1.4 times higher for women reporting high PSS (OR = 1.36, 95% CI 1.02‐1.81) (Table 5). Comparison with the constant‐only model, however, indicated that the correct classification percentage was unchanged (63.7%), suggesting a limited contribution of PSS.

#### 3.5.2 Block 2 (PSS and epidural)

The model remained significant (χ^2^[*df* = 2] = 178.94, *P* < .001). Epidural was uniquely associated with augmentation (Wald[1] = 150.36, *P* < .001); women receiving an epidural were almost 10 times more likely to also have augmentation (OR = 9.75, 95% CI 6.78‐14.03) (Table 5). PSS was rendered nonsignificant (Wald[1] = 0.66, *P* = .418), with epidural use accounting fully for the observed association between PSS and augmentation in labor.

### 3.6 Indirect effects

**FIGURE 2:**
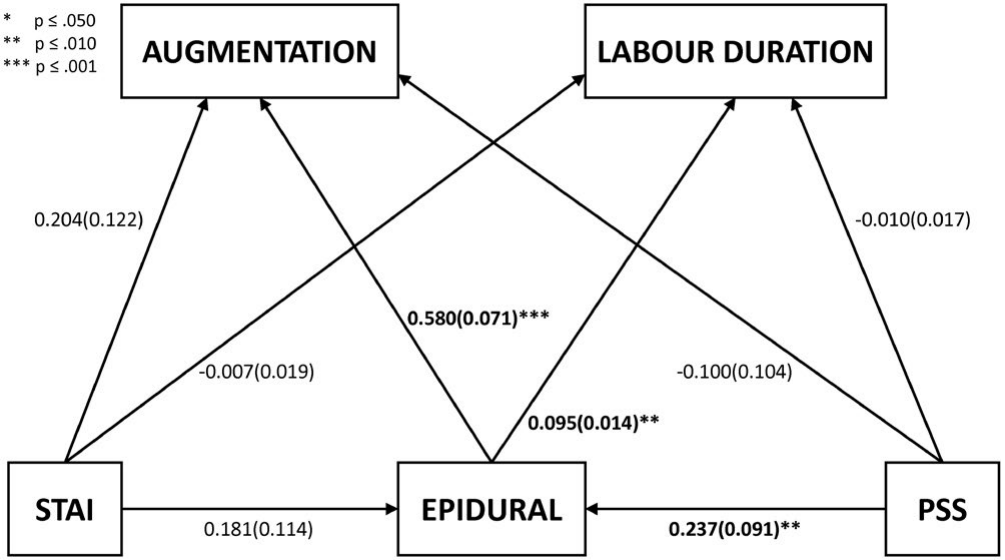
Path analysis, coefficients reported as *b*(SE). PSS, pregnancy‐specific stress; STAI, State‐Trait anxiety inventory

Given the pattern of effects that emerged in relation to the first two hypotheses, a path analysis model was run (Figure 2) to investigate the presence of indirect effects, leading from anxiety scores to labor duration and to augmentation use, through epidural.

Notably, in the context of this broader model, all effects related to STAI scores were rendered nonsignificant, whereas PSS scores showed a direct pathway to epidural but not augmentation.

Two significant indirect paths emerged from this model: first, elevated PSS scores were found to increase indirectly the likelihood of augmentation, through the increase in the likelihood of epidural use (*b*[SE] = 0.137 (0.056), 95% Bias Corrected CI 0.035‐0.261, *P* = .015). Secondly, a similar pattern emerged in relation to labor duration, with an increase in this being related to elevated PSS scores, through the mediation of increased epidural use (*b*[SE] = 0.022 (0.009), 95% Bias Corrected CI = 0.006‐0.042, *P* = .015).

## 4 DISCUSSION

This paper presents one of the few large‐sample studies considering the associations between antenatal anxiety and labor duration and associated obstetric interventions with appropriate controls for confounding variables. It is important to recognize that despite popular belief, neither anxiety nor pregnancy‐specific stress was directly associated with a longer first stage of labor duration. This confirms the finding by Reck et al^7^ for generic anxiety but differs for pregnancy‐specific stress. In the current study, pregnancy‐specific stress was assessed at 20 weeks of gestation, whereas Reck et al^7^ assessed a similar construct at 33 weeks of gestation. The timing of assessment is contentious, since stress nearer to childbirth may be a more potent predictor of obstetric outcome, but for screening purposes an earlier assessment is needed to enable psychological intervention. For Reck et al,^7^ fear of giving birth was the strongest predictor of labor duration (*R* ^2^ = .13). In contrast, in the present study, the pregnancy‐specific stress measure focused on feelings (eg feeling scared) about pregnancy, not birth. Large‐scale studies are needed to consider the specific role of FOC in relation to birth duration.

Initial associations between general anxiety and pregnancy‐specific stress and augmentation became nonsignificant after epidural use was controlled for. Only one other study has investigated the association between antenatal anxiety and augmentation^6^ and that reported higher rates of augmentation in women with FOC. However, any potential mediation by epidural was untested. Augmentation of labor is important as it may negatively influence women’s experiences of childbirth.^10^, ^27^ A key question is therefore whether anxiety leads to a woman having an epidural, which subsequently increases the likelihood of requiring augmentation, or vice versa. Certainly, the path analysis supports the former and clinically this is a commonly observed progression. However, slow labor is also exhausting and the use of augmentation increases the pain of contractions considerably. In clinical practice, therefore, it is also not unusual for an epidural to be administered at the same time as augmentation is started. Path analysis cannot prove causation and merely demonstrates that these data are consistent with the former model of understanding.

A key finding was that whereas both general anxiety and pregnancy‐specific stress showed similar patterns in their association with labor duration, epidural and augmentation‐only pregnancy‐specific stress demonstrated the specific indirect pathway via an increased likelihood of receiving an epidural, for both augmentation and longer labor duration. Pregnancy‐specific stress, being more focused, may be better linked with sustained anxiety in pregnancy and more evident in the birth context. Women with elevated fear or anxiety during pregnancy are more likely to receive an epidural during labor.^6^, ^10^ In turn, there is a consistent association between epidural and longer first stage of labor and assisted birth.^28^ Few studies have examined a potential indirect pathway between antenatal anxiety/stress with augmentation or first stage labor duration via receipt of epidural.^6^, ^29^ Adams et al^6^ reported that labor duration was longer for women with FOC, and that women with FOC were more likely to receive an epidural. When both FOC and epidural were entered into a regression model predicting labor duration, the magnitude of association between FOC and duration was attenuated but remained significant. FOC is also a narrower construct than pregnancy‐specific stress.

Although assessment of pregnancy‐specific stress at 20 weeks of gestation will not necessarily identify women likely to experience longer labor, it may identify those with greater likelihood of requiring epidurals. Epidurals provide significant benefits in pain relief, but women perhaps need to be more aware they may influence labor progression and subsequent need for augmentation. In addition, there may be benefits in identification of pregnancy‐specific anxiety and offering psychological interventions to enhance birth experience and subsequent postnatal mental health. Interestingly, a randomized controlled trial of universal provision of self‐hypnosis in pregnancy led to reductions in fear and anxiety experienced in childbirth but did not reduce epidural rate,^30^ so intervention targeting may be of value.

The large, representative sample recruited from the Wirral Peninsula sole provider for prenatal care, and the use of standardized tools assessing anxiety and pregnancy‐specific stress are strengths. However, it is important to note that the broader construct of pregnancy‐specific stress was measured rather than FOC, which is possibly more pertinent. In addition, only the STAI 'state' form rather than the 'trait' version was used and it could be that the latter would have produced different findings. STAI anxiety levels, as would be expected in a consecutive sample, were unremarkable, although PSS scores were relatively low.^20^, ^21^, ^31^ Further information about distributions is published in previous papers from the WCHADS study.^32^ Labor duration was defined as the onset of regular painful contractions to full dilation of the cervix; merging definitions for latent and first stage of active labor. Women with elective cesareans or with emergency cesarean section when not in labor were obviously excluded as no duration of labor could be measured, because labor was not experienced. However, elective cesarean section rates in this service were very low, as this was only carried out for specific medical reasons, thus the sample would have represented the full range of PSS scores. These findings relate only to the first stage of labor and not total labor duration. This paper does not examine prediction of mode of birth.

It must be noted that the data were for a consecutive sample of primiparous women collected in 2008/2009. Although rates of interventions may differ at different times and localities, the important feature of this work is the identification of a particular pathway of effect, which is unlikely to be time‐ or place‐specific.

## 5 CONCLUSION

Early assessment of antenatal state anxiety or pregnancy‐specific stress did not directly aid prediction of the duration of the first stage of labor. Pregnancy‐specific stress but not general anxiety was particularly linked to both longer labor and augmentation via indirect pathways through epidural. Pregnancy‐specific stress rather than general anxiety may need to be the focus of any psychological screening in pregnancy. Interventions for pregnancy‐specific stress now need systematic testing with psychological measures and epidurals as outcomes.

## CONFLICT OF INTERESTS

None.
